# Laccase Immobilized on a PAN/Adsorbents Composite Nanofibrous Membrane for Catechol Treatment by a Biocatalysis/Adsorption Process

**DOI:** 10.3390/molecules19033376

**Published:** 2014-03-19

**Authors:** Qingqing Wang, Jing Cui, Guohui Li, Jinning Zhang, Dawei Li, Fenglin Huang, Qufu Wei

**Affiliations:** Key Laboratory of Eco-Textiles, Ministry of Education, Jiangnan University, Wuxi 214122, China; E-Mails: wqq888217@126.com (Q.W.); wykaojn@126.com (J.C.); leeanna101121 @yeah.net (G.L.); qq164542493@126.com (J.Z.); ldw19900323@163.com (D.L.); windhuang325@163.com (F.H.)

**Keywords:** laccase, enzyme immobilization, adsorbent, catechol, adsorption

## Abstract

The treatment of catechol via biocatalysis and adsorption with a commercial laccase immobilized on polyacrylonitrile/montmorillonite/graphene oxide (PAN/MMT/GO) composite nanofibers was evaluated with a homemade nanofibrous membrane reactor. The properties in this process of the immobilized laccase on PAN, PAN/MMT as well as PAN/MMT/GO with different weight ratios of MMT and GO were investigated. These membranes were successfully applied for removal of catechol from an aqueous solution. Scanning electron microscope images revealed different morphologies of the enzyme aggregates on different supports. After incorporation of MMT or MMT/GO, the optimum pH showed an alkaline shift to 4, compared to 3.5 for laccase immobilized on pure PAN nanofibers. The optimum temperature was at 55 °C for all the immobilized enzymes. Besides, the addition of GO improved the operational stability and storage stability. A 39% ± 2.23% chemical oxygen demand (COD) removal from the catechol aqueous solution was achieved. Experimental results suggested that laccase, PAN, adsorbent nanoparticles (MMT/GO) can be combined together for catechol treatment in industrial applications.

## 1. Introduction

Laccase, a multicopper oxidase, has been widely used in various applications including paper manufacturing [1], wood processing [2], environmental bioremediation [3,4], food industry [5], as well as textile engineering [6]. With the increasing demand, laccase products have been customized for specific industrial applications. Though the high quality laccase production process has been improved over the last decades, industrial application of laccase is still hampered by a lack of long-term operational stability and the difficulty in recycling laccase. 

Enzyme immobilization techniques are well recognized as a common way to overcome the drawbacks mentioned above. They provides a more convenient handling of the enzyme, facilitate its facile separation from the products, minimize or eliminate protein contamination of the product, exhibit low or no allergenicity, and facilitate efficient recovery, and reuse of the enzyme, thus enabling its cost-effective use in continuous operation. Aside from this, they provide generally enhanced stability under both storage and operational conditions [7]. Many different kinds of immobilization methods have been reported for laccase immobilization, for instance, entrapment, encapsulation, adsorption, covalent binding, self-immobilization, and different combinations of the aforementioned methods [8]. Among all those methods, adsorption is a relatively simple and inexpensive way to immobilize laccase and may therefore have a higher commercial potential than other methodologies [9]. The adsorption of laccase onto a support is based on ionic and/or other weak forces of attraction. The pH and ionic strength of the medium and the hydrophobicity of the support surface must be taken into account during the immobilization process [10]. Some studies have shown that adsorption is preferable to other techniques for the immobilization of the laccase from *T. versicolor* [8,11].

As is known, there is no universal support surface for immobilization of all kinds of enzymes. The support should be insoluble and compatible with laccase, without unfavorable interactions between the enzyme and the support. Besides, the diffusion limitations should be minimized to facilitate the biocatalytic reaction [12]. Electrospun nanofibers have been considered as an ideal support due to their high surface area to volume ratio, high porosity and the interconnectivity of the electrospun nanofibers, which endows them with a low hindrance for mass transfer [13]. Polyacrylonitrile (PAN) nanofibers have been widely studied for enzyme immobilization due to their good mechanical properties, solvent resistance, abrasion resistance, and high tensile strength [14,15,16,17,18]. A very recent work reported by Xu *et al.* [16] used direct conjugation of laccase molecules onto the surface of chemically modified PAN electrospun nanofibers and the performance of the immobilized laccase in removing 2,4,6-trichlorophenol was investigated. The results showed that the operational properties and the storage stability of the immobilized enzymes were greatly improved. Gupta and Dhakate [18] immobilized lipase on electrospun PAN nanofiber membrane by both physical adsorption and covalent bonding. The lipase immobilized by physical adsorption showed higher transesterification and hydrolytic activities than that covalently linked or native lipase. All this makes the use of PAN nanofibrous membrane as a support to immobilize enzymes by physical adsorption seem a very promising and feasible process. 

In this study, we adopted an easy and feasible adsorption method for the direct immobilization of the commercial laccase onto the surface of PAN, PAN/MMT, PAN/MMT/GO composite electrospun nanofibrous membranes without using any chemical modification. Since the PAN used in this work was obtained from an industrial product, which was polymerized with a second monomer (methyacrylate) and a third monomer (itaconic acid). The use of itaconic acid was expected to contribute reactive groups to improve the multipoint attachment between enzyme and the support. Besides, the MMT and GO nanolayers could adsorb the laccase reaction products and their properties in the oxidation and removal of catechol from aqueous solutions were investigated.

## 2. Results and Discussion

### 2.1. Morphologies of MMT, GO, MMT/GO Composites, and PAN/MMT/GO Composite Nanofibers

**Figure 1:**
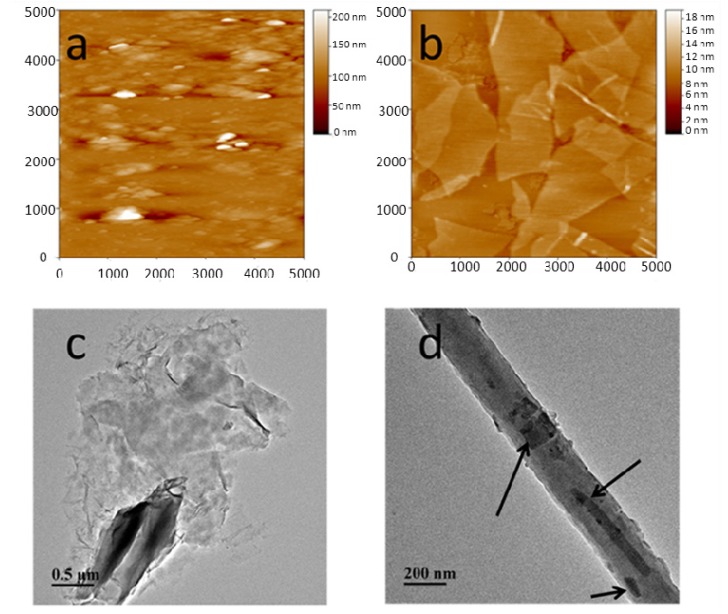
(**a**) tapping mode topography of MMT. (**b**) tapping mode topography of GO. (**c**) TEM of MMT/GO complexes. (**d**) TEM of PAN/MMT/GO-2 composite nanofibers.

The topographies of MMT and GO are shown in Figure 1a,b. The pure MMT aggregates showed a spherical shape and had an average particle size of 85.6 nm, while the GO showed a layered sheet structure with a fractal shape extended to more than 3 μm. Compared with MMT, the dimensions of GO were much larger than those of MMT aggregates, so after blending, MMT was wrapped in GO sheets, as presented in Figure 1c. For PAN/MMT/GO composite nanofibers, some nanoparticles can be clearly observed within the polymer matrix, as seen in Figure 1d.

### 2.2. Relationship between Enzyme Structure and Activity

**Figure 2:**
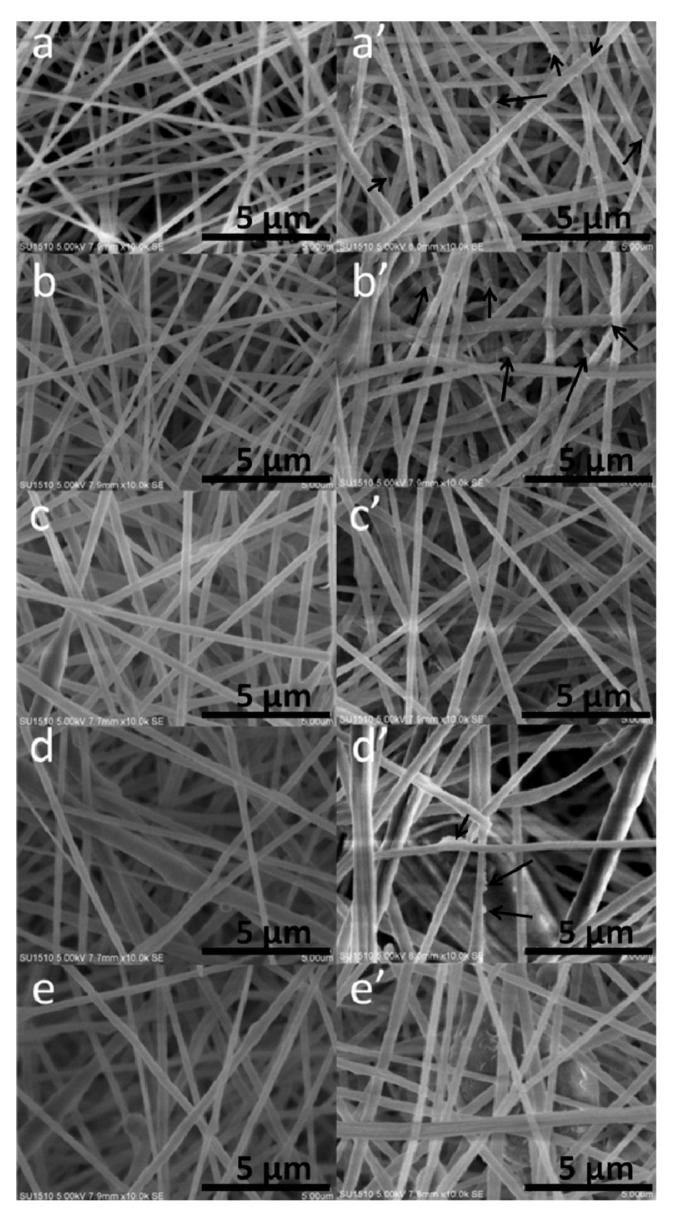
SEM images of PAN, PAN/MMT, PAN/MMT-GO composite nanofibers before and after enzyme immobilization: (**a** and **a’**) 0 wt. % MMT; (**b** and **b’**) 5 wt. % MMT; (**c** and **c’**) 5 wt. % MMT/GO, MMT:GO = 9:1; (**d** and **d’**) 5 wt. % MMT/GO, MMT:GO = 8:2; (**e** and **e’**) 5 wt. % MMT/GO, MMT:GO = 7:3.

The SEM images of the nanofibrous membrane before and after enzyme immobilization are displayed in Figure 2. Before enzyme immobilization (see Figure 2a–e), the surface of the nanofibers was uniform. Compared with the pure PAN (see Figure 2a), the diameter of the composite nanofibers increased after the incorporation of the nanoparticles (see Figure 2b). With the addition of GO, beaded structures can be found in the fibrous structure.

**Figure 3:**
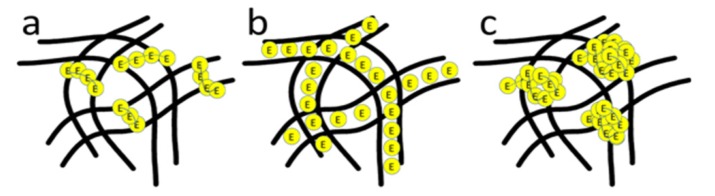
Three types of enzyme immobilization morphology (**a**) strip-shape. (**b**) uniform coating membrane. (**c**) particle aggregates.

After enzyme immobilization (see Figure 2a’–e’), the nanofibers showed an increased fiber diameter due to the swelling behavior during the enzyme immobilization process. The immobilized enzyme aggregates formed different morphologies on the surface of the nanofibers, which can be divided into three types, *i.e.*, strip-like structures, uniformly coated membrane [19,20], and also particle aggregates [21], illustrated schematically in Figure 3. This phenomenon might be caused by different enzyme-support linkages. 

It was noticeable that the immobilized enzymes on PAN and PAN/MMT composite nanofibers formed an extraordinary strip-like structure and twined or half-twined around the nanofibers, which hadn’t been reported before as far as we are aware. After addition of GO, the morphology of the immobilized laccase changed into a uniform coating structure. However, for PAN/MMT/GO-2, the case was a little bit different. Apart from the uniform coating, the immobilized laccase also presented an aggregated particle structure.

**Figure 4:**
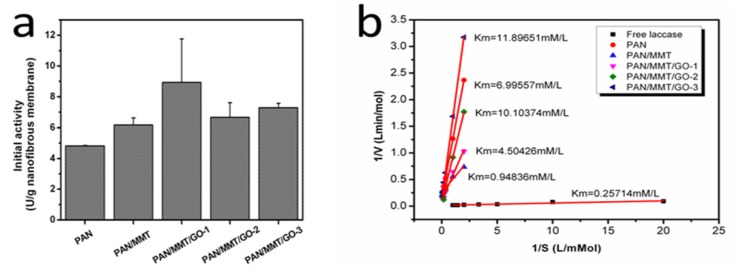
The activities of immobilized laccase on PAN, PAN/MMT, PAN/MMT/GO composite nanofibers in an aqueous buffer solution (pH 4.5, temperature 30 °C).

The activities of PAN-Lac, PAN/MMT-Lac, PAN/MMT/GO-1-Lac, PAN/MMT/GO-2-Lac, PAN/MMT/GO-3-Lac are presented in Figure 4a. Compared with PAN-Lac, the activities of the other four were relatively higher, which can be attributed to the nanoparticles incorporated inside the polymer matrix. Besides, the PAN/MMT/GO-1-Lac showed the highest activity, which can be partially explained by the improved enzyme loading of laccase, as revealed in the SEM analyses (Figure 2) combined with schematic illustrations (Figure 3). After enzyme immobilization, the enzyme diffusion rate and variations in the microenvironment was changed, leading to the loss of catalytic activity. *K_m_* values of the free and immobilized enzyme were revealed by Figure 4b. 

The *K_m_* values of the immobilized laccase were significantly higher than that of free laccase, which was due to the lower accessibility between substrate and active points of the immobilized enzyme caused by space barriers of the supports and the increased diffusion limitation [22]. Among those immobilized enzymes, PAN/MMT showed lowest *K_m_*, which can be attributed to the adsorption properties of MMT [23]. With the addition of GO, the MMT was wrapped and the adsorption properties were weakened, leading to an increased *K_m_*.

### 2.3. Immobilized Enzyme Properties (pH, Temperature, Storage, Reusability)

**Figure 5:**
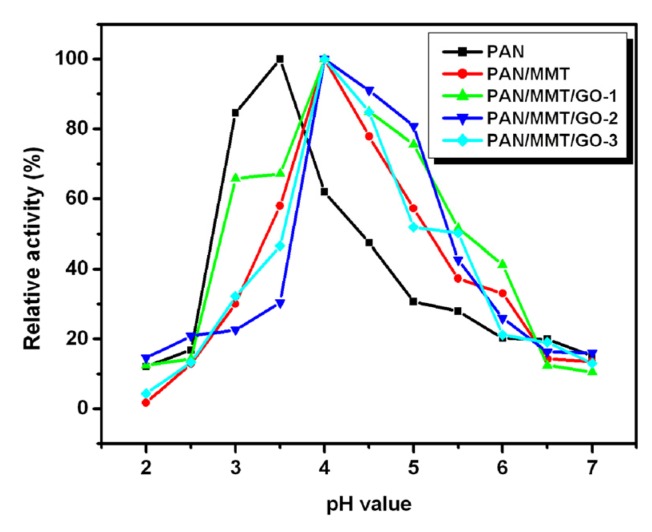
Optimum pH of the immobilized laccase on different supports.

It can be seen that the activity of the immobilized enzyme is greatly dependent on pH (Figure 5). Changes in pH values could affect the enzyme conformation and the degree of dissociation the of the substrate, and thus the binding and catalysis effects between the enzyme molecules and substrate were also influenced. At a specific pH value the most appropriate combination between enzyme and the substrate can happen, resulting in a highly efficient catalysis. The laccase immobilized on PAN nanofibers showed a maximum activity at pH 3.5, whereas the laccase immobilized on the composite nanofibers was most active at pH 4. This could be explained by the cationic ion adsorption capability of the MMT. Some H^+^ would gather around the nanofiber’s surface, especially those areas there MMT exists, so the pH around the composite nanofiber was considered more acidic than that of the pure PAN at the same buffer solution, which finally leads to the right shift of the optimum pH value.

**Figure 6:**
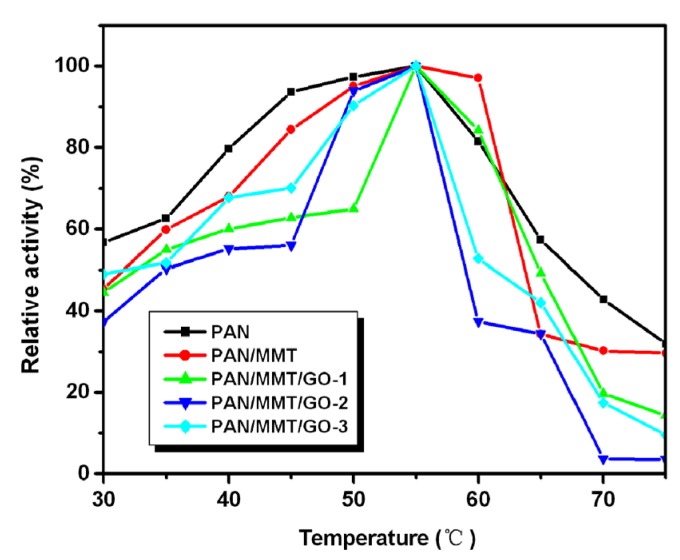
Optimum temperature of immobilized laccase on different suppports.

The influence of temperature on the activity of the immobilized laccase is shown in Figure 6. The immobilized enzymes were incubated in buffer (pH 4.5) for 5 min at different temperatures varying from 30 to 75 °C before adding ABTS. 

The immobilized enzymes showed a similar trend of temperature stability in the range of 30–75 °C, while the laccase immobilized on PAN and PAN/MMT nanofibers showed relatively higher activity stability than the laccase immobilized on those nanofibers with GO, which may be at least in part due to the strip-like structure. All the immobilized enzymes showed relatively higher enzyme activity at the 45–60 °C range, with an optimum temperature at 55 °C. 

**Figure 7:**
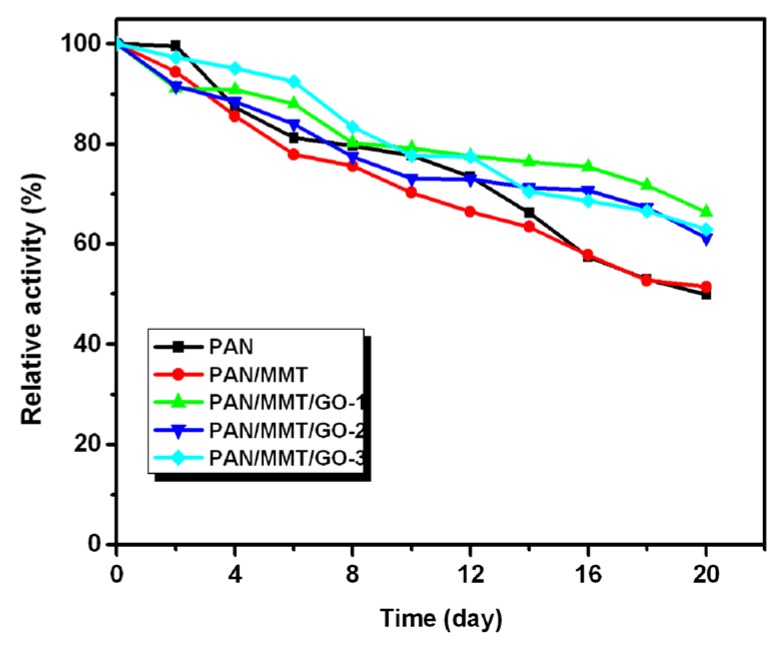
Storage stability of immobilized laccase on different supports.

The storage stability of the immobilized enzymes was also studied and the results are presented in Figure 7. The laccase immobilized on PAN/MMT/GO composite nanofibers showed relatively higher storage stability than that of PAN and PAN/MMT. The immobilized enzymes retained more than 50% of their original activity after 20 days.

**Figure 8:**
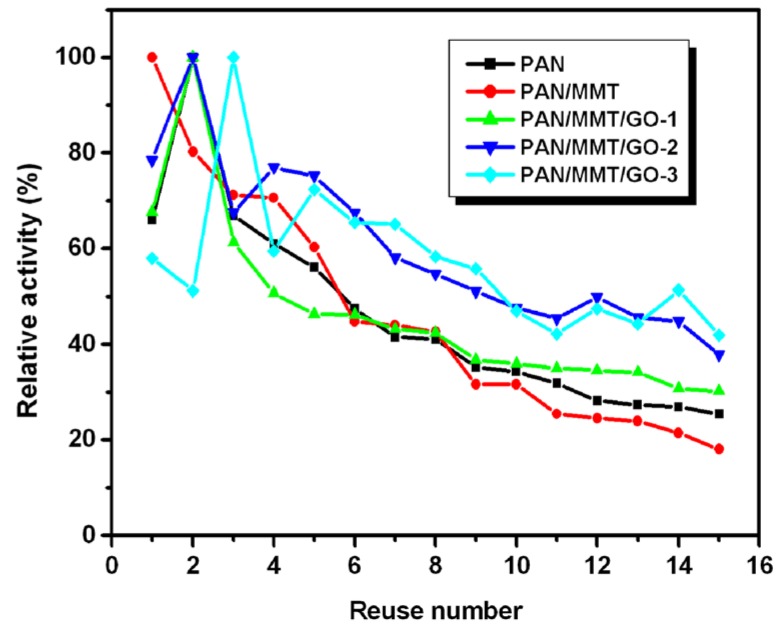
Operational stability of the immobilized laccase on different supports.

The operational stability of the immobilized laccase is presented in Figure 8. Reusability of immobilized enzyme is considered to be the most important in terms of industrial applications, because repeated use can reduce the production cost. The immobilized enzyme retained more than 50% of its initial activity after five repeated recycles. 

Decrease in the enzyme activity upon repeated usage was expected due to the fact that enzymes might denature during the operation process. However, for enzymes immobilized on PAN and PAN/MMT/GO nanofibrous membrane, the case was different. As is seen in Figure 8, the enzymes reached their highest activity at the second or third cycle. This phenomenon can be attributed to the flexible texture of the nanofibrous membrane. The membrane became loose and fluffy during repeated usage, contributing to more sites for the enzyme to reach the substrate. With the increase of GO concentration, the operational stability was improved. PAN/MMT/GO-3, after being used 15 times, retained 72% of the initial activity.

### 2.4. Catechol Treatment by a Homemade Membrane Reactor Treatment

**Figure 9:**
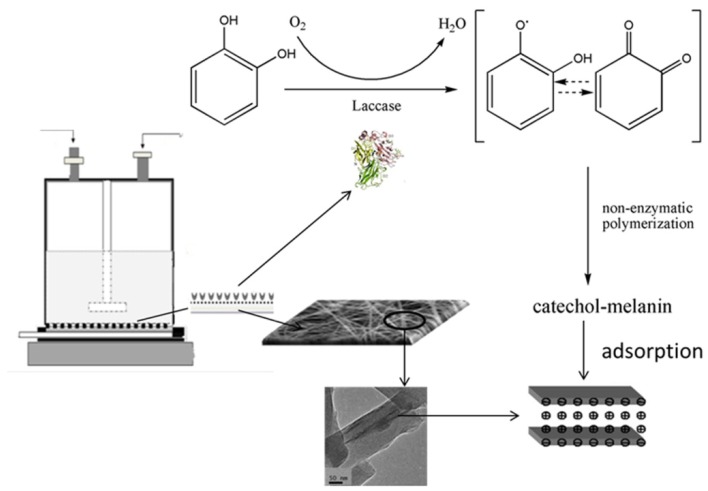
Schematic illustration of the homemade membrane reactor for catechol treatment.

An ultrafiltration device was used here as a membrane reactor, but instead of a micro-filtration membrane/ultra-filtration membrane (MF/UF), the electrospun nanofibrous membrane were used. As shown by the schematic illustration (Figure 9), laccase can catalyze the biotransformation of catechol into quinone, which then undergoes a series of non-enzymatic polymerization reactions leading to the formation of catechol-melanin complexes [24], which can be further adsorbed by MMT.

**Figure 10:**
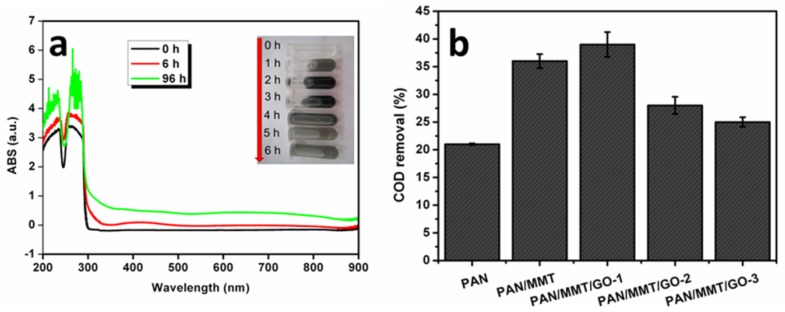
(**a**) UV-vis spectra of end products from catechol catalyzed by laccase immobilized on PAN/MMT, the inset shows digital photographs of the solutions collected at different time intervals; (**b**) Catechol COD removal by the immobilized enzyme.

The UV-vis spectra showed that after laccase treatment, a peak at 410 nm which is due to the formation of quinone can be seen (Figure 10a). The end product solution first became darker and then changed to be clear again (Figure 10a inset), indicating the simultaneous occurrence of both quinone polymerization to form catechol-melanin and adsorption by MMT. The COD removal of the catechol solution by the laccase immobilized on the composite membranes is presented in Figure 10b. The PAN/MMT/GO-1 showed better COD removal capability than PAN/MMT. As is known, MMT can adsorb catechol [25], and those adsorbed catechol molecules can be protected from being transformed by laccase [26]. Since the overall adsorption capacity has been determined by the MMT itself, in this case, less catechol-melanin can be further adsorbed. After addition of GO, the process of catechol adsorption by wrapped MMT was slowed down, leading to a more complete catalytic reaction by laccase. The adsorption of catechol-melanin by MMT decreased the COD of the end solution. Further addition of GO showed a decreased COD removal ratio, which was due to the fact that less MMT was incorporated.

## 3. Experimental

### 3.1. Chemicals

Commercial laccase (3 U/mg) powder from *Trametes versicolor* was purchased from Wuhan Nuohui Pharmaceutical and Chemical Co., Ltd. (Wuhan, China). 2,2'-Azino-bis-(3-ethyl-benzothiazoline-6-sulfonic acid, ABTS) was obtained from Richu Biosciences Co., Ltd. (Shanghai, China). GO was purchased from XF Nano, Inc. (Nanjing, China). MMT organically modified with hexadecyltrimethyl ammonium bromide (CTAB) was supplied by Zhejiang Fenghong Clay Chemicals Co., Ltd (Zhejiang, China). PAN (Mw = 50,000 g mol^−1^) was obtained from Shangyu Wu & Yue Economic and Trade Co. Ltd (Zhejiang, China). All other reagents were of analytical grade and were purchased from Sinopharm Chemical Reagent Co., Ltd (Shanghai, China). 

### 3.2. Preparation of Electrospun PAN, PAN/MMT and PAN/MMT/GO Composite Nanofibers

First, MMT and GO with an total weight of 150 mg were dispersed in DMF by repeated stirring and sonication for about 3 h. Then PAN powder (3 g) was added into the solution and magnetically stirred for about 24 h until a homogeneous solution was obtained. Then the solutions were electrospun at a positive voltage of 15 kV with a working distance of 15 cm, and a flow rate of 0.5 mL/h. The as-prepared electrospun composite nanofibers from solutions with different MMT/GO weight ratios were denoted as PAN/MMT-GO-1 (MMT:GO = 9:1), PAN/MMT-GO-2 (MMT:GO = 8:2), PAN/MMT-GO-3 (MMT:GO = 7:3).

### 3.3. Immobilization of Laccase

The laccase was dissolved in acetic acid/sodium acetate buffer (pH = 4.5) solution at a concentration of 3 g/L by magnetic stirring for 20 min in ice bath. The supernatant was collected by centrifuging for 5 min and used for the following immobilization process. The nanofibrous membrane (100 mg, accurately weighed) was placed into centrifuge tubes and then enzyme solution (8 mL per tube) was distributed into them. The immobilization process was conducted in the refrigerator at 4 °C for 12 h. After that, the membranes were taken out and washed thoroughly with buffer solution until no enzyme can be detected in the washing solution.

### 3.4. Determination of Immobilized Laccase Activity

The immobilized enzyme activity was assayed at 30 °C using ABTS as the substrate. The detailed process was reported in our previous paper [27]. Three replications of all assays were conducted.

Kinetic tests were carried out at 30 °C in 100 mM sodium acetate (pH = 4.5) buffer using ABTS as the substrate, with the substrate concentration varied from 0.1 to 1 mM. The kinetic parameters of *K_m_* and *V_max_* were calculated according to the Lineweaver-Burk double reciprocal models [28].

To determine the optimum pH, the immobilized enzymes were incubated in buffers with pH ranging from 2 to 7 at 4 °C for 12 h and then assayed for activity, while the optimum temperature was determined by the activities of the immobilized enzymes incubated in buffers (pH 4.5) for 5 min at different temperatures varying from 30 to 75 °C before adding ABTS.

The storage stability of the immobilized enzyme was determined by the activity retention ratio during storage at 4 °C in 100 mM sodium acetate buffer solution (pH 4.5), at a regular intervals up to 20 days. 

The operational stability was studied by repeated usage for 15 times, and the relative enzyme activity was recorded. The experiments were carried out at 30 °C, pH 4.5. All the control samples were made with the buffer solution with the same pH value as the assayed one.

### 3.5. Catecholl Degradation

The catechol degradation process was carried out by our homemade membrane reactor. Instead of the MF/UF membrane, nanofibrous membrane after enzyme immobilization was used. The catechol powders were dissolved in buffer solution (pH 4) at a final concentration of 5 mM. Then, catechol solution (300 mL) was added into the bottle and magnetically stirred to achieve a more uniform reaction.

### 3.6. Characterizations

An atomic force microscope (AFM, Benyuan CSPM 4000, Guangzhou, China) was used in this work to observe the surface morphology of the MMT/GO hybrids. The samples were prepared by applying one droplet of the MMT or GO solution to the surface of a freshly peeled mica slip, and drying in an oven under 40 °C. All the AFM images were obtained in the tapping mode.

A Hitachi H-7500 transmission electron microscope (TEM, Tokyo, Japan) was used to examine the assembly behavior of the MMT/GO hybrids and also the morphology of the resulting polymer matrix. The MMT/GO hybrids with the weight ratio of 8:2 and PAN/MMT/GO-2 composite nanofibers were chosen for this study. The experiments were operated under the voltage of 80 kV.

The morphology of the electrospun nanofibers before and after enzyme immobilization was characterized by scanning electron microscope (SEM, Quanta 200, Holland FEI Company, Beijing, China). The samples were sputter coated with a thin layer of Au nanoparticles to reduce the charging effects.

## 4. Conclusions

PAN, PAN/MMT, and PAN/MMT/GO composite nanofibers were prepared by an electrospinning process and their properties as supports were examined by immobilizing laccase onto the fibers’ surface. The immobilized laccase showed distinct morphologies on different supports. The activity of the immobilized laccase could reach as high as 8.7 U/g nanofibrous membrane. The K_m_ value for PAN/MMT was small. The operation stability and storage stability were improved after the addition of GO. This membrane was further used in a homemade reactor for catechol treatment. The COD removal reached 39% ± 2.23%. This treatment method is simple, low cost and produces no secondary pollution, making it a good candidate for future industrial applications.
